# Effects of Applied Voltages on the Charge Transport Properties in a ZnO Nanowire Field Effect Transistor

**DOI:** 10.3390/ma13020268

**Published:** 2020-01-07

**Authors:** Jongwon Yoon, Fu Huang, Ki Hoon Shin, Jung Inn Sohn, Woong-Ki Hong

**Affiliations:** 1 Jeonju Center, Korea Basic Science Institute, Jeonju-si, Jeollabuk-do 54907, Korea; jwyoon@kbsi.re.kr (J.Y.); hf3546@kbsi.re.kr (F.H.); 2 Division of Physics and Semiconductor Science, Dongguk University-Seoul, Seoul 04620, Korea; kihoonshin@dongguk.edu

**Keywords:** ZnO, nanowire, charge transport, field effect transistor, conduction mechanism

## Abstract

We investigate the effect of applied gate and drain voltages on the charge transport properties in a zinc oxide (ZnO) nanowire field effect transistor (FET) through temperature- and voltage-dependent measurements. Since the FET based on nanowires is one of the fundamental building blocks in potential nanoelectronic applications, it is important to understand the transport properties relevant to the variation in electrically applied parameters for devices based on nanowires with a large surface-to-volume ratio. In this work, the threshold voltage shift due to a drain-induced barrier-lowering (DIBL) effect was observed using a Y-function method. From temperature-dependent current-voltage (I-V) analyses of the fabricated ZnO nanowire FET, it is found that space charge-limited conduction (SCLC) mechanism is dominant at low temperatures and low voltages; in particular, variable-range hopping dominates the conduction in the temperature regime from 4 to 100 K, whereas in the high-temperature regime (150–300 K), the thermal activation transport is dominant, diminishing the SCLC effect. These results are discussed and explained in terms of the exponential distribution and applied voltage-induced variation in the charge trap states at the band edge.

## 1. Introduction

Zinc oxide (ZnO) has received considerable interest over the past few decades as a promising material for a variety of applications in electronics, optics, and photonics because it exhibits a direct wide bandgap (~3.37 eV), a large exciton binding energy (60 meV), a variety of nanoscale forms, and piezoelectricity [1,2]. Recently, ZnO nanostructures have attracted much attention to the fields of nanoscale electronic and optoelectronic devices, such as sensors [3], solar cells [4], energy harvesting devices [5], light-emitting diodes [6], and especially field effect transistors (FETs) [7].

Since the FET based on nanowires is one of the fundamental building blocks in potential nanoelectronic applications, it is very important to understand charge transport behaviors in nanowire-based transistors. The electrical properties of nanowire-based FET devices sensitively depend on their size and shape, defects and impurities, and surface states or defects [7,8,9]. Moreover, it has been generally accepted that the contacts between the nanowire and the metal electrodes play also an important role in the charge transport properties of nanowire-based FETs due to their large surface-to-volume ratio coupled with unique geometry [10,11,12]. For example, Lee and coworkers reported the distinct electrical transport features of FETs made from ZnO nanowires with two different types of geometric properties: one type consisted of corrugated nanowires with a relatively smaller diameter and higher density of surface states or defects, and the other type involved smooth ZnO nanowires with a relatively larger diameter and lower density of surface states or defects [7]. Lord et al. [10] showed that the electrical transport behavior of nanocontacts between ZnO nanowires and Au metals can switch from Schottky to Ohmic depending on the size of the metal contact in relation to the nanowire diameter. Jo et al. [11] and He et al. [12] demonstrated the influence of the contact resistance on the electrical properties in In_2_O_3_ and ZnO nanowires, respectively.

In addition to structural geometry effects associated with nanowires and devices, importantly, a better understanding of the charge transport properties relevant to the variation in the electrical parameters actually applied to devices based on nanowires is required for the application of new nanoscale electronics and devices. Recently, several studies on the effect of bias stress in ZnO nanowire FETs have been reported [13,14]. Ju et al. [13] reported the effects of bias stress (gate or drain stress) on the stability of the ZnO nanowire FET with a self-assembled organic gate insulator. Choe et al. [14] investigated the threshold voltage instability induced by gate bias stress in ZnO nanowire FETs, which is associated with the trapping of charges in the interface trap sites located in interfaces between the nanowire and dielectric layer.

Herein, we report the effect of applied gate and drain voltages on the charge transport properties in a ZnO nanowire FET with a back-gated configuration. To do this, temperature-dependent current-voltage (I-V) measurements from 4 to 300 K were carried out. Using a *Y*-function method, we find that the threshold voltage (V_th_) shifts to a negative gate bias direction due to the drain-induced barrier lowering (DIBL) effect, leading to increasing carrier concentration in the channel. The temperature-dependent I-V measurements show that the transport behavior of the fabricated ZnO nanowire FET is governed by space charge-limited conduction (SCLC) at low temperatures and low voltages, in particular by variable-range hopping (VRH) conduction mechanism in the temperature regime from 4 to 100 K, and by the thermal activation transport at the high-temperature regime (150–300 K).

## 2. Materials and Methods

**Figure 1:**
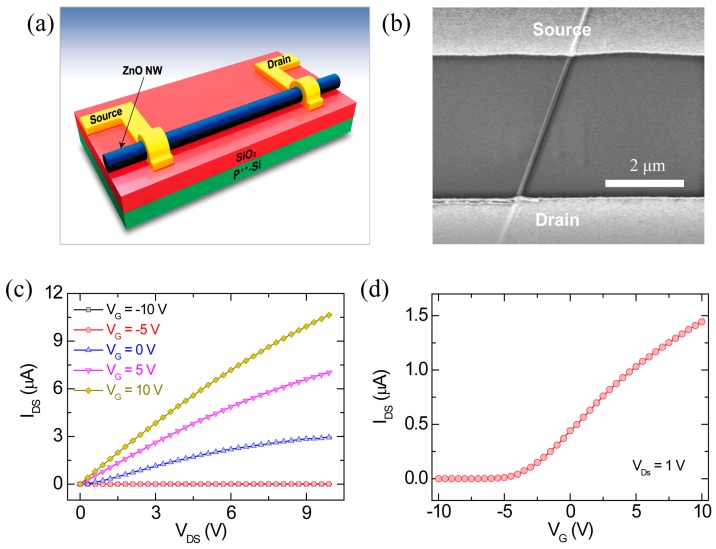
(**a**) Schematic illustration of the fabricated ZnO nanowire FET with a back-gate configuration; (**b**) A SEM image of the fabricated ZnO nanowire FET; (**c**) Output characteristics (I_DS_-V_DS_) and (**d**) transfer characteristics (I_DS_-V_G_) at V_DS_ = 1 V of the fabricated ZnO nanowire FET, which was measured at room temperature. The inset in (**d**) shows a semi-logscale I_DS_-V_G_ curve at V_DS_ = 1 V.

High-density ZnO nanowires were grown on Au-coated c-plane sapphire substrates by a vapor transport method without using metal-catalysts. To grow the high-density ZnO nanowires, a mixed source of ZnO powder (99.995%) and graphite powder (99%) in a ratio of 1:1 was blended with ethanol. The source materials and substrates were placed in an alumina boat, which was then loaded into the center of a horizontal tube furnace. The furnace was heated at a rate of 35 °C/min and held at approximately 920 °C for 40–60 min. During the whole growth process, a mixed gas of Ar and O_2_ with mixture ratio of 99:1 was maintained and then the flow rate of the mixed gas was 20 SCCM (standard cubic centimeters per minute) and the pressure of the furnace was kept at approximately 600 Torr. When the furnace was allowed to cool to room temperature naturally, a large amount of a white product was grown on the surface of the Au-coated c-plane sapphire substrate (not shown). Structural characterization of the ZnO nanowires vertically grown on the sapphire substrate was performed using field emission scanning electron microscope (FESEM) and transmission electron microscope (TEM), as shown in Figure S1. The energy dispersive x-ray spectroscopy (EDS) of the as-grown ZnO nanowires shows compositional elements (the inset in Figure S1a). The TEM images (Figure S1c–e) indicate that the growth direction of the ZnO nanowires is along the c-axis. A selected area electron diffraction (SAED) pattern confirms the (0001) growth direction (the inset of Figure S1d). The photoluminescence (PL) measurement of the ZnO nanowires at room temperature was examined by utilizing a FEX system (NOST, Seongnam-si, Korea) with a He–Cd laser (325 nm) as an incident excitation source (Figure S2). Next, the ZnO nanowires that were grown on the Au-coated sapphire substrate were transferred onto a highly-doped silicon wafer with 100 nm-thick thermally grown silicon dioxide (SiO_2_) by dropping and drying a liquid suspension of ZnO nanowires for the fabrication of FET devices. For all the fabricated ZnO nanowire FETs, source and drain electrodes consisting of Ti (100 nm)/Au (80 nm) were deposited by an electron beam evaporator, as shown in Figure 1a. The distance between the source and drain electrodes is approximately 4 μm (Figure 1b). The electrical properties of the nanowire FET device were characterized using a semiconductor characterization system (Keithley 4200-SCS, Keithley, Cleveland, OH, USA) at a temperature range of 4–300 K. It should be noted that even though the nanowires are synthesized in the same conditions, there can be wire-to-wire or device-to-device variations in the electrical and optical properties, which strongly depend on the dimension (diameter and length, etc.) and surface states of the as-grown nanowires [7,15].

## 3. Results and Discussion

A schematic illustration and a scanning electron microscopy (SEM) image of the fabricated ZnO nanowire FET with a back-gate configuration are shown in Figure 1a,b. Figure 1c,d shows the output (I_DS_-V_DS_) and transfer (I_DS_-V_G_) characteristics of the fabricated ZnO nanowire FET with a back-gate configuration (Figure 1a,b), respectively. The fabricated ZnO nanowire FET showed typical n-type semiconductor properties and depletion-mode operation, which exhibited a nonzero current at zero gate bias and a negative threshold voltage [15].

**Figure 2:**
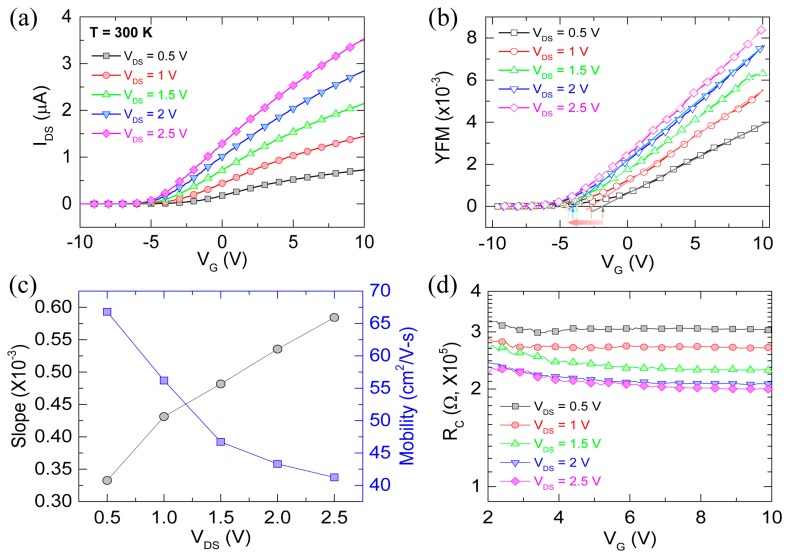
(**a**) I_DS_-V_G_ curves measured at room temperature (T = 300 K) for the ZnO nanowire FET, with V_DS_ varying from 0.5 to 2.5 V; (**b**) YFM value as a function of V_G_ at different V_DS_ values for the ZnO nanowire FET. From the linear fitting, *V_th_* and mobility can be extracted from the V_G_-axis intercept and the slope, respectively. Each arrow indicates the *V_th_* for each V_DS_; (**c**) Slope and mobility as a function of V_DS_ extracted from linearly fitted curves in (**b**); (**d**) Contact resistance as a function of gate bias, with V_DS_ varying from 0.5 to 2.5 V.

Figure 2a shows the transfer characteristics at different drain-source voltages for the fabricated ZnO nanowire FET measured at room temperature. From this, electrical characteristics were analyzed by the *Y*-function method (YFM) (Figure 2b), which has been widely used for contact resistance and mobility based on a straightforward analysis of the drain current (I_DS_) in the linear region (electron accumulation region) [16,17]. The Y-function can be obtained from the I_DS_-V_G_ (Figure 2a) as follows [17],

(1) $$Y=\frac{I_{DS}}{\sqrt{g_{m}}}=\sqrt{V_{DS}\frac{\mu C_{G}}{L^{2}}}\left(V_{G}-V_{th}\right)$$

where *g_m_* = *dI_DS_*/*dV_G_*, *μ* is the mobility, *C_G_* is the gate capacitance, *L* is the channel length, and *V_th_* is the threshold voltage, in which *μ* and *V_th_* can be determined from the slope and the *V_G_*-axis intercept of the linear region of the *Y*-function, respectively (Figure 2b,c). In Figure 2b, it is clearly seen that *V_th_* shifts to a negative gate bias direction (marked by arrows) when *V_DS_* increases from 0.5 to 2.5 V, which indicates the DIBL effect [18]. This effect can reduce the Schottky barrier between source/drain electrodes and the nanowire contacts, affecting the contact resistance (*R_C_*). Using the *Y*-function, the *R_C_* at interfaces between source/drain electrodes and the ZnO nanowire can be calculated from the following equation [17],

(2) $$R_{C}=R_{tot}-R_{ch}=\frac{V_{DS}}{I_{DS}}-\frac{V_{DS}}{k^{2}\left(V_{GS}-V_{th}\right)}$$

where *k* is the slope of the linear region of the *Y*-function. The slopes of the linear region of the *Y*-function are different (Figure 2c), indicating the difference in *R_C_* [17] (Figure 2d). Importantly, the contact resistance is present at a metal-nanowire interface and can affect the electrical performance of nanowire FETs [19]. The work function difference between the ZnO and the contact metal leads to the formation of an energy barrier at the interface between the two materials, which can influence the barrier height.

**Figure 3:**
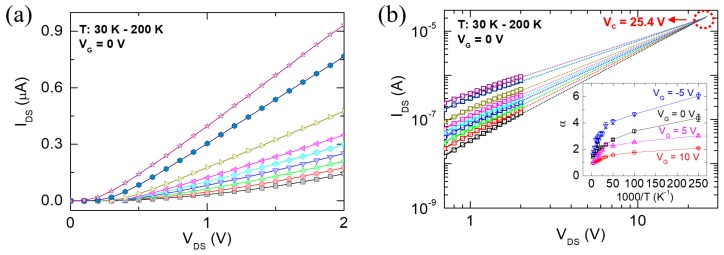
(**a**) I_DS_-V_DS_ curves measured at V_G_ = 0 V and different temperatures (30–200 K) for the ZnO nanowire FET; (**b**) The extrapolation derived from the corresponding logscale I_DS_-V_DS_ characteristics at different temperatures of (**a**), which provide a critical voltage (*V_C_*).

To understand the charge transport mechanism in our nanowire FET with different contact resistances, the temperature-dependent electrical measurement and analyses of the ZnO nanowire FET were examined. Figure 3a shows the I_DS_-V_DS_ characteristics of the ZnO nanowire FET at different temperatures ranging from 30 to 200 K. With decreasing temperature, the I_DS_ decreased, indicating a strong temperature dependence. In addition, the logscale I_DS_–V_DS_ showed the power law relationship, I ∝ V^α^, and such power law dependence with α > 2 is a characteristic feature of SCLC in a semiconductor with an exponential charge trap distribution at the band edge [19,20]. The exponents, α, were extracted from logscale I_DS_-V_DS_ curves in the temperature range from 4 to 300 K at different gate biases, as shown in the inset of Figure 3b. The α values increased with decreasing temperature, exceeding 2 in the low-temperature range. This result implies the existence of trap states in the ZnO nanowire. The values reached approximately 1 in the high- temperature range due to the thermally activated electrons, resulting in deviation from SCLC. The trap densities (*N_t_*) can be estimated by extrapolating the logscale I_DS_-V_DS_ characteristics, as shown in Figure 3b. Figure 3b shows a crossover point at which the conductance was independent of the temperature. The V_DS_ value at the crossover point is denoted as a crossover voltage (*V_c_*) and it was approximately 25.4 V. The *V_c_* can be expressed by [20],

(3) $$V_{C}=\frac{qN_{t}L^{2}}{2\varepsilon_{0}\varepsilon_{r}}$$

where *q* is the electric charge, *L* is the channel length, *ε_0_* is the vacuum permittivity, and *ε_r_* is the relative permittivity of ZnO (~8.5). From the above equation, the calculated *N_t_* at *V_c_* = 25.4 V, was 1.5 × 10^15^ cm^−3^. According to previous reports [21,22], most of the trap densities arise from oxygen vacancies located on the nanowire surface rather than at the nanowire center. Therefore, the calculated *N_t_* may correspond to the interface trap states at the metal-nanowire contacts or the nanowire-dielectric layer, which could affect the charge transport of the ZnO nanowire FET.

**Figure 4:**
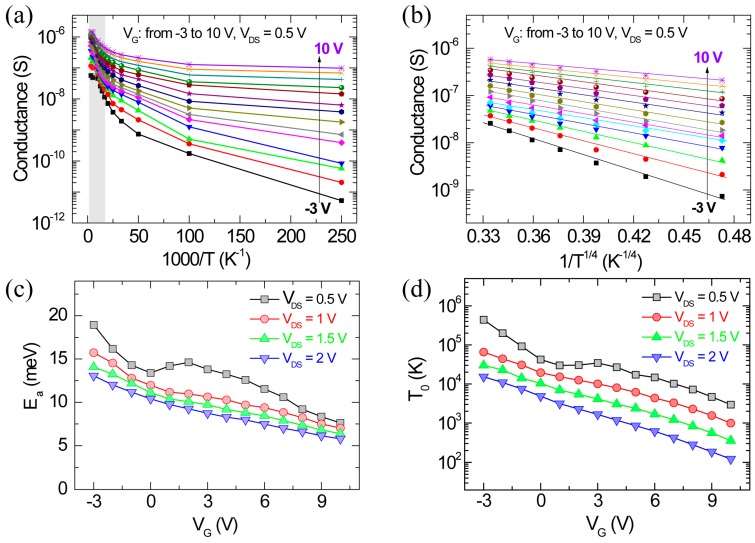
(**a**) Arrhenius plots of the conductance (*G*) versus *1000/T* at different gate voltages from −3 to 10 V for V_DS_ = 0.5 V. (**b**) Semilogarithm plots showing the temperature dependence of conductance (*G*) vs *1/T^1/4^* fitted by Equation (5) at different gate voltages for V_DS_ = 0.5 V. The activation energy (*E_a_*) (**c**) and characteristic temperature (*T_0_*) (**d**) depending on the applied gate and drain voltages.

Next, we carried out analyses of the Arrhenius plots of the conductance (*G*) versus 1000/T at different *V_G_* values to further investigate the transport mechanism of the ZnO nanowire FET, as shown in Figure 4a,b. Two different regimes in the temperature-dependent conductance of the nanowire FET device were clearly observed at different V_G_ values (V_G_ from −3 to 10 V, 1 V steps), implying different charge transport mechanisms. Note that the Arrhenius plots at low V_DS_ regime (0.5, 1, 1.5, and 2 V) were also characterized for different V_G_ values. In the high-temperature region (150–300 K) (marked by the gray-colored region), the thermally activated carriers were dominant in the charge transport, indicating a conductance proportional to *exp(−E_a_/kT)*, which can be expressed as Equation (4) below [23,24,25].

(4) $$G=G_{0}exp\left(-\frac{E_{a}}{k_{B}T}\right)$$

where *G* and *G_0_* are the conductance and weak temperature-dependent constant, respectively, *E_a_* is the activation energy, *k_B_* is the Boltzmann constant, and *T* is the temperature. The *E_a_* characterized by the linear region in the semi-log plot of conductance versus *1/T* is shown in Figure 4a. Here, the *E_a_* can be extracted by the linear fits in the high-temperature region in Figure 4a (marked by the gray-colored region). Figure 4c shows the extracted *E_a_* as a function of the V_G_ at different V_DS_ values for the device. The *E_a_* decreased due to the lowered Schottky barrier at the metal/semiconductor interface when the applied biases increased, including V_G_ and V_DS_. In contrast, in the low-temperature region (4–100 K), the carrier conduction is mainly attributed to VRH, which exhibits charge transport through the trap states near the Fermi level. According to previous reports [23,26,27,28,29,30], the VRH conduction can be expected due to charge trapping at localized states in semiconducting nanomaterials at low applied bias and low temperature where the Fermi level lies in localized sates within a band gap. The conductance following the three-dimensional (3D) VRH mechanism can be expressed by the following equation [25,31,32],

(5) $$G=G_{0}exp\left[-{\left(\frac{T_{0}}{T}\right)}^{1/4}\right]$$

where *T*_0_ are the characteristic characteristic temperature. Figure 4b shows that the low-temperature conductance of the device is well fitted by the 3D VRH as a function of *T*^−1/4^ at low applied bias, indicating that the conductance follows 3D VRH model well for low electric fields. From Equation (5), the values of *T*_0_, which represent how actively VRH occurs [25,31,32], were extracted, as shown in Figure 4d. As the applied biases (V_G_ and low V_DS_) increased, the *T*_0_ also continuously decreased, implying reduced VRH conduction. The result might be due to the enhanced electron concentration from the lowering of the Schottky barrier. The increased electron concentration might additionally fill the trap states, leading to the reduction in hopping conduction [25,31,32]. As a result, the *E_a_* and *T*_0_ values can be modified by the applied electric field, which is associated with the modulation of localized trap states. This trend is consistent with the results reported for semiconducting nanomaterials with localized trap states [23,30,33].

**Figure 5:**
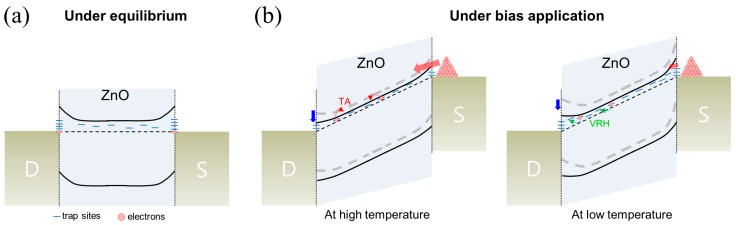
Energy band diagrams depicting the charge transport mechanism for the ZnO nanowire FET (**a**) under equilibrium and (**b**) under bias application at low and high temperatures. The blue arrow indicates Schottky barrier modulation according to the applied gate and drain voltages.

The energy band diagram presented in Figure 5 qualitatively shows the charge transport mechanisms of the ZnO nanowire FET, as discussed above. Unlike the equilibrium condition (Figure 5a), the applied biases (V_G_ and V_DS_) could induce Schottky barrier modulation, resulting in changes in the carrier injection properties at the metal-semiconductor contact, as shown in Figure 5b. As a result, the modified Schottky barrier could affect the carrier concentration, leading to a change in the density of localized trap states in the channel. Furthermore, different temperature-dependent charge transport mechanisms were observed. Specifically, thermal activated (TA) conduction of electrons from a shallow level of localized states was dominant for charge transport in the high-temperature range, denoted as TA in Figure 5b (left), whereas the VRH conduction through the trap states near the Fermi level was dominant in the low-temperature range, denoted as VRH in Figure 5b (right).

## 4. Conclusions

In summary, we fabricated a ZnO nanowire FET with a back-gated configuration and characterized the electrical properties of the FET device through temperature-dependent measurements to study the effect of applied gate and drain voltages on the charge transport properties. The Y-function method showed that the *V_th_* shifted to a negative gate bias direction due to the DIBL effect. The temperature-dependent I-V measurements showed that the transport behavior of the ZnO nanowire FET was governed by SCLC at low temperatures and low voltages, in particular, by VRH conduction in the temperature regime from 4 to 100 K and by thermal activation transport at the high-temperature regime (150–300 K).

## Supplementary Materials

The following are available online at https://www.mdpi.com/1996-1944/13/2/268/s1, Figure S1: SEM and TEM characterizations of the as-grown ZnO nanowires, Figure S2: PL data of the as-grown ZnO nanowires.
