# Supplementary material for: Effects of Applied Voltages on the Charge Transport Properties in a ZnO Nanowire Field Effect Transistor
Source: Materials (Basel). 2020 Jan 7;13(2):268. doi: 10.3390/ma13020268 (PMC7014215; doi:10.3390/ma13020268)
Supplement: Supplementary file 1 [file materials-13-00268-s001.pdf]

materials

MDPI

Supplementary Materials

Effects of Applied Voltages on the Charge Transport Properties in a ZnO Nanowire Field Effect Transistor

Jongwon Yoon [1], Fu Huang [1], Ki Hoon Shin [2], Jung Inn Sohn [2,*] and Woong-Ki Hong [1,*]

[1] Jeonju Center, Korea Basic Science Institute, Jeonju-si, Jeollabuk-do 54907, Korea; jwyoon@kbsi.re.kr (J.Y.); hf3546@kbsi.re.kr (F.H.)
[2] Division of Physics and Semiconductor Science, Dongguk University-Seoul, Seoul 04620, Korea; kihoonshin@dongguk.edu
* Correspondence: junginn.sohn@dongguk.edu (J.I.S.); wkh27@kbsi.re.kr (W.-K.H.)

Received: 20 November 2019; Accepted: 31 December 2019; Published: 7 January 2020

The grown ZnO nanowires were characterized by scanning electron microscope (SEM) and transmission electron microscope (TEM), as shown in Figure S1. The average diameter of ZnO nanowires was approximately 89.4 nm and the composition ratio was 1:1 for Zn and O from energy dispersive spectroscopy (EDS). The TEM image also showed that the as-grown nanowires were single crystals and the growth direction was along the c-axis.

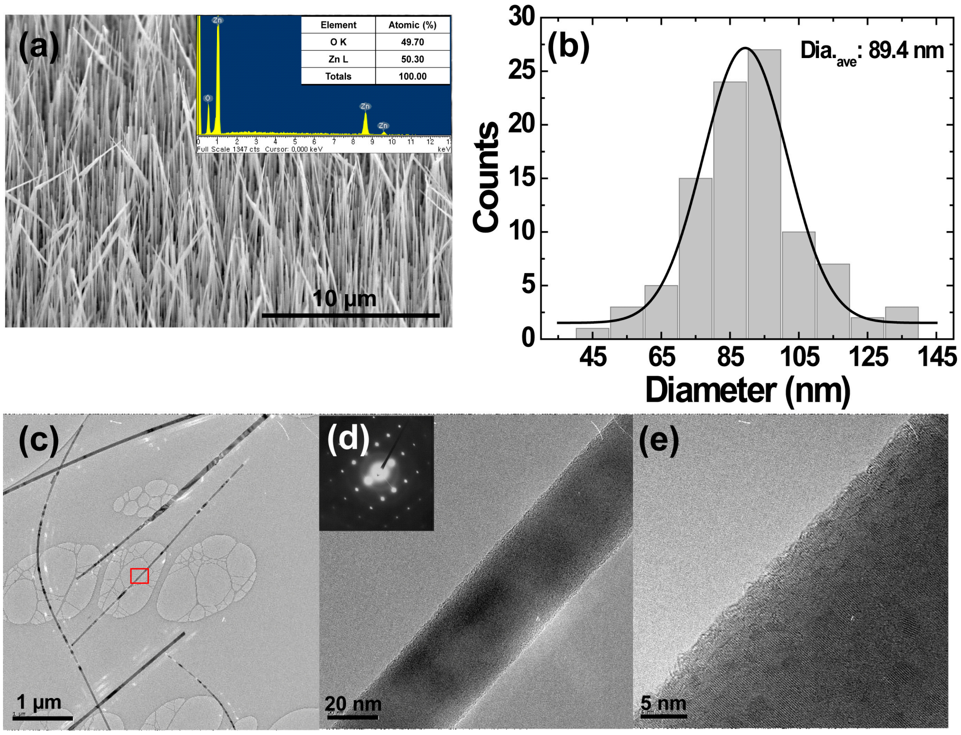

Figure S1. (**a**) SEM image of the ZnO nanowires vertically grown on a sapphire substrate. The inset shows the energy dispersive x-ray spectroscopy (EDS) data of the as-grown ZnO nanowires. (**b**) Diameter distribution of the as-grown ZnO nanowires. (**c–d**) Low magnification TEM images of ZnO nanowires. The inset shows the SAED pattern of the ZnO nanowire. (**e**) High magnification TEM image of the ZnO nanowire.

Figure S2 show the PL spectra measured at room temperature for the as-grown ZnO nanowires. The PL emissions of the ZnO nanowires consist of the sharp near-bandedge (NBE) emission band with a peak position between 375 and 380 nm and the broad defect-related emission (or trap-state

emission) in the visible range, which is a surface-related process [1]. In Figure S2, the defect emission of the as-grown ZnO nanowires is strong, indicating large density of surface states and/or defects. Such surface states and/or defects can affect the charge transport properties of the ZnO nanowire-based transistor devices [1].

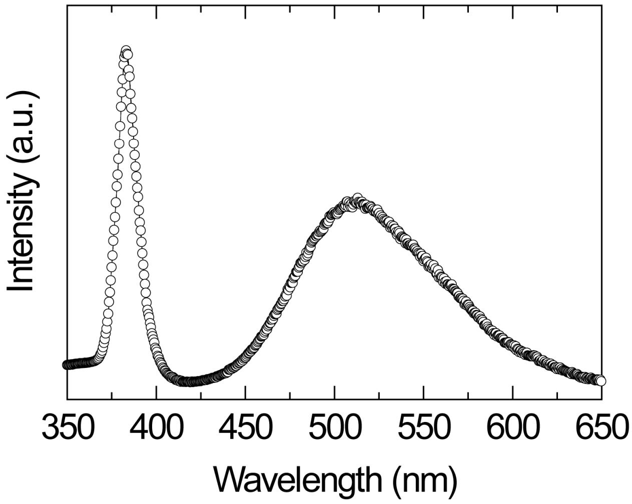

Figure S2. PL data of the as-grown ZnO nanowires

Reference

1. Hong, W.-K.; Sohn, J.I.; Hwang, D.-K.; Kwon, S.-S.; Jo, G.; Song, S.; Kim, S.-M.; Ko, H.-J.; Park, S.-J.; Welland, M.E.; Lee, T. Tunable electronic transport characteristics of surface-architecture-controlled zno nanowire field effect transistors. *Nano Lett.* **2008**, *8*, 950–956.
